# Are glucose levels, glucose variability and autonomic control influenced by inspiratory muscle exercise in patients with type 2 diabetes? Study protocol for a randomized controlled trial

**DOI:** 10.1186/s13063-016-1156-0

**Published:** 2016-01-20

**Authors:** ASO Schein, APS Correa, Karina Rabello Casali, Beatriz D. Schaan

**Affiliations:** Exercise Pathophysiology Research Laboratory, Hospital de Clínicas de Porto Alegre, Porto Alegre, Rio Grande do Sul Brazil; Faculty of Health Sciences, University of Sydney, Lidcombe, New South Wales Australia; Institute of Science and Technology, Universidade Federal de São Paulo, São Paulo, Brazil; Graduate Program in Endocrinology, Universidade Federal do Rio Grande do Sul, Porto Alegre, Rio Grande do Sul Brazil; Endocrine Division, Hospital de Clínicas de Porto Alegre, Porto Alegre, Rio Grande do Sul Brazil

**Keywords:** breathing exercises, exercise, glucose, type 2 diabetes

## Abstract

**Background:**

Physical exercise reduces glucose levels and glucose variability in patients with type 2 diabetes. Acute inspiratory muscle exercise has been shown to reduce these parameters in a small group of patients with type 2 diabetes, but these results have yet to be confirmed in a well-designed study. The aim of this study is to investigate the effect of acute inspiratory muscle exercise on glucose levels, glucose variability, and cardiovascular autonomic function in patients with type 2 diabetes.

**Methods/design:**

This study will use a randomized clinical trial crossover design. A total of 14 subjects will be recruited and randomly allocated to two groups to perform acute inspiratory muscle loading at 2 % of maximal inspiratory pressure (PImax, placebo load) or 60 % of PImax (experimental load).

**Discussion:**

Inspiratory muscle training could be a novel exercise modality to be used to decrease glucose levels and glucose variability.

**Trial registration:**

ClinicalTrials.gov NCT02292810.

## Background

Hyperglycemia is the main cause of the clinical manifestations and acute and chronic complications of diabetes mellitus. Reducing glucose levels through nonpharmacological [1, 2] and pharmacological therapy is the cornerstone of type 2 diabetes management [3, 4]. Goals for optimal diabetes management include reducing glycated hemoglobin (HbA1c) levels to 7 % and implementing a number of cardiovascular risk reduction strategies [5, 6].

However, chronic complications of diabetes can develop even when these targets are met. This can be attributed to genetic and epigenetic factors [7], disruption of repair mechanisms [8], and, possibly, high glucose variability even when optimal HbA1c levels are achieved. Glucose variability is the fluctuation of glucose levels during the day, including episodes of hypoglycemia and hyperglycemia [9]. Measurement of glucose levels in intersticium throughout the day by continuous glucose monitoring systems [10, 11] or periodic capillary blood-glucose measurements [12, 13] have been used to evaluate glucose variability. Several conventional measures are available to quantify this parameter and have been widely reported in the literature over the past years [14, 15], including the mean amplitude of glycemic excursions [16], the standard deviation of glucose, and the mean of daily differences [17]. Non-conventional methods, which use mathematical and statistical analysis of the dynamic characteristics of glucose levels, as measured using continuous glucose monitoring systems, through spectral analysis and symbolic analyses, were recently reported [18].

Exercise training, consisting of aerobic exercise, resistance exercise, or a combination of both, is a useful tool for reducing HbA1c levels in type 2 diabetes [2, 19, 20]. It is also associated with several other benefits, including improved blood pressure control [21], reduction of skeletal muscle frailty [22], reduction of inflammation [23], and improved cardiovascular autonomic function [24]. Additionally, a recent study showed that an acute bout of aerobic or resistance exercise is associated with lower glucose variability [18], opening a new venue of benefits to explore.

Considering that many diabetic patients, especially those with autonomic neuropathy, have poor exercise tolerance or are completely unable to perform conventional exercises [25], alternatives should be sought. Inspiratory muscle training is a modality that has proven benefits in several clinical situations [26, 27], such as improving cardiopulmonary capacity and normalizing derangements in autonomic function [27, 28]. In patients with type 2 diabetes, this exercise modality can improve the strength and resistance of inspiratory muscles [29] and reduce glucose levels and glucose variability [30]. We speculate that, in type 2 diabetes, beyond reflecting the well-known improvement in insulin sensitivity induced by muscle contractions, inspiratory exercise would also yield improvements in cardiovascular autonomic function [27, 28]. Thus, the aim of this study is to investigate the effect of acute inspiratory muscle exercise on glucose levels, glucose variability, and autonomic control in patients with type 2 diabetes.

## Methods/design

### Study design

This study is a randomized clinical trial with a crossover design.

### Outcome measures

The difference in glucose levels before and after exercise (evaluated by continuous glucose monitoring systems; glucose level after exercise minus glucose level before exercise) is the primary outcome measure. The secondary outcome measures are: (1) changes in glucose variability, which will also be evaluated by continuous glucose monitoring systems through conventional (mean amplitude of glucose excursion, variation of glucose, coefficient of variation of glucose, and standard deviation of glucose) and non-conventional indices (spectral analysis and symbolic patterns: no variation); (2) changes in cardiovascular autonomic control, which will be evaluated by blood pressure variability and heart rate variability (measures of power spectral density of low frequency, high frequency and very low frequency bands). Table 1 shows the measures that will be evaluated to achieve the desired results.

**Table 1:** Evaluated measures

Time point	Data collection	Measure	Outcomes
Pre-evaluation	Blood and urine analysis	Glycemia, HbA1c, microalbuminuria and creatinine	Metabolic responses
	Cardiovascular autonomic assessment	Ewing tests	Diagnosis of cardiovascular autonomic neuropathy
	International Physical Activity Questionnaire	Survey	Physical activity estimates
	Inspiratory muscle test	maximal inspiratory mouth pressure (PImax)	Inspiratory muscle strength
	Pulmonary function test	maximum voluntary ventilation, forced vital capacity, vital capacity, forced expiratory volume (1 s), forced expiratory volume (1 s)/vital capacity and forced expiratory volume (1 s)/forced vital capacity	Pulmonary flow and volume
	Rest electrocardiogram	12-lead electrocardiogram	Electrical activity of the heart
	Cardiopulmonary exercise testing	VO_2peak_, VCO_2peak_, R_peak_, VE_peak_, VE⁄VO_2peak_, VE⁄VCO_2peak_	Ventilatory and metabolic variables
At rest	Continuous glucose monitoring system	Glucose concentration	Glucose levels and variability
	Biopac system	Continuous recording of blood pressure	Blood pressure and heart rate variability in time and frequency domains
Controlled ventilation	Continuous glucose monitoring system	Glucose concentration	Glucose concentration and variability
	Biopac system	Continuous recording of blood pressure	Blood pressure and heart rate variability in time and frequency domains
Inspiratory muscle exercise (2 % or 60 % of maximal inspiratory mouth pressure, PImax)	Continuous glucose monitoring system	Glucose concentration	Glucose levels and variability
	Biopac system	Continuous recording of blood pressure	Blood pressure and heart rate variability in time and frequency domains
Recuperation	Continuous glucose monitoring system	Glucose concentration	Glucose concentration and variability
	Biopac system	Continuous recording of blood pressure	Blood pressure and heart rate variability in time and frequency domains

HbA1c, glycated hemoglobin; PImax, inspiratory mouth pressure; R, respiratory exchange rate; VCO_2_peak: carbon dioxide output; VE, minute ventilation; VO_2peak_, peak oxygen uptake; VE⁄VO_2_: ventilatory equivalent for oxygen; VE⁄VCO_2_: ventilatory equivalent for carbon dioxide

### Sample size and power calculation

According to preliminary results obtained in our laboratory [31], the sample size was estimated in 14 individuals with type 2 diabetes, allowing for a dropout rate of 10 %. This sample size would detect a 34 mg/dl difference in glucose levels between the intervention inspiratory load of 60 % and the placebo inspiratory load of 2 %, taking into account a standard deviation of 30 mg/dl, a statistical power of 80 %, and a type I error rate of 5 %. WinPepi software was used to calculate the sample size.

### Inclusion and exclusion criteria

The study will be conducted in patients with type 2 diabetes, diagnosed according to the National Diabetes Data Group criteria [5]. The inclusion criteria will be age 30 years and older, HbA1c between 7.5 and 10 %, and stable clinical condition, to ensure safe performance of the trial protocols. Patients will be recruited from the Outpatient Endocrinology Clinic of Hospital de Clínicas de Porto Alegre, Brazil. All study methods described are in accordance with the CONSORT Statement [32].

The exclusion criteria will consist of: insulin treatment, pregnancy, pulmonary disease (history of exercise-induced asthma), cardiac arrhythmias, chronic kidney disease (glomerular filtration rate <30 ml/min), exclusive use of beta blockers as antihypertensive therapy, current smoking, varicose veins, and musculoskeletal conditions that would hinder safe completion of the proposed exercise protocols.

### Randomization

The randomization sequence will be generated by computer. Randomization to the low-resistance inspiratory muscle exercise group (2 % of maximal inspiratory pressure (PImax), placebo load) or the high-resistance inspiratory muscle exercise group (60 % of PImax, experimental load) will be performed by an investigator assigned exclusively to this task, who will not participate in the recruitment, assessment, or intervention phases of the study.

### Data collection

Patients will be instructed to attend on three different days at the study laboratory for baseline evaluations, as follows:

- Day 1: Urine collection and fasting blood draw for assessment of glucose, HbA1c, creatinine, and microalbuminuria; physical examination; Ewing tests for autonomic assessment; and completion of the International Physical Activity Questionnaire.
- Day 2: Resting 12-lead electrocardiogram, inspiratory muscle and pulmonary function tests.
- Day 3: Cardiopulmonary exercise testing.

### Autonomic neuropathy evaluation

The presence of cardiovascular autonomic neuropathy will be evaluated using digital electrocardiography (VNS-Rhythm Neurosoft, Ivanovo, Russia) as previously described [33] and the five noninvasive cardiovascular tests proposed by Ewing [34], as standardized in our institution [35]. The heart rate, electrocardiographically monitored, will be evaluated after 5 min of resting and before and after deep breathing, the Valsalva maneuver, and standing. Three tests will be used to evaluate the heart rate response to (1) deep breathing, (2) lying-to-standing heart rate ratio, and (3) a Valsalva maneuver. Two tests of blood pressure control will be performed during (1) orthostatic hypotension and (2) sustained handgrip. Two or more abnormal test results will be deemed diagnostic of cardiovascular autonomic neuropathy. Heart rate variation will be assessed while patients are asked to breathe deeply at a rate of six breaths per minute while being monitored using three-lead electrocardiography. The maximum and minimum heart rate during each breathing cycle will be measured, and the mean difference over six cycles will be calculated. The heart rate ratio test will be performed after a period of rest in the supine position, and heart rate variability will be determined by calculating the maximal to minimal heart rate ratio: the ratio of the longest RR interval, measured around the 30th beat after standing up, to the shortest RR interval, measured around the 15th beat after standing up. The Valsalva maneuver will consist of forced exhalation into a mouthpiece with a pressure of 40 mmHg for 15 s, and the ratio of the maximum RR interval after this maneuver to the minimum RR interval during the maneuver will be calculated. Orthostatic hypotension will be defined as a decrease of 30 mmHg in systolic blood pressure when the individual voluntarily changes from the supine to the standing position. Measurements will be obtained at minutes 1 and 3 after standing up. Sustained muscle contraction will be measured by a handgrip dynamometer. The dynamometer will be first squeezed to maximum isometric contraction in the dominant hand. The highest of three measurements will be taken; then patients will be asked to hold at 30 % maximum for 3 min. A rise in diastolic blood pressure of < 10 mmHg is considered an abnormal response.

### Inspiratory muscle testing

Inspiratory muscle function testing will be performed using a pressure transducer (MVD-500 V.1.1 Microhard System; Globalmed, Porto Alegre, Brazil), connected to a system containing two one-way valves (DHD Inspiratory Muscle Trainer, Chicago, IL, USA) and equipped with a 2 mm diameter orifice to relieve facial muscle pressure. Patients will be instructed to remain seated, with elbows resting on the chair; a nasal clip will be used during all maneuvers. The PImax will be determined after patients take a deep breath at tidal volume, followed by slow exhalation to residual volume, and then quickly perform a maximal inspiration against the occluded circuit with a small air leak. The test will be repeated at least three times, with a 1 min interval between repetitions, to obtain six measurements with <10 % variation [29, 36, 37]. The values obtained from the PImax readings will be verified directly on the transducer. Predicted values will be corrected for age and sex.

### Pulmonary function

Measurements of forced vital capacity, vital capacity, forced expiratory volume in 1 s, and their ratios (forced expiratory volume (1 s)/vital capacity and forced expiratory volume (1 s)/forced vital capacity) will be obtained using a computerized spirometer (Eric Jaeger GmbH, Würzburg, Germany), as recommended by the American Thoracic Society [38], and results will be expressed as a percentage of predicted parameter. For maximum voluntary ventilation, subjects will be instructed to breathe as deeply and quickly as possible for 15 s; volume measurement will begin after the patient is able to achieve and maintain maximum effort [38].

### Cardiopulmonary exercise testing

Maximal functional capacity will be assessed by means of an incremental exercise test to be performed on a treadmill (Inbramed 10200, Porto Alegre, Brazil), using a ramp protocol with a speed of 2–6 km/h and increasing slope of 4–10 % to reach exhaustion in about 10 min [36]. A 12-lead electrocardiogram tracing will be obtained every 1 min (Nihon Khoden Corp., Tokyo, Japan), and blood pressure will be measured every 2 min with a standard cuff sphygmomanometer. During the test, gas exchange variables will be measured breath-by-breath with a previously validated system (Metalyzer3B, CPX System; Cortex, Leipzig, Germany). Ventilatory and metabolic variables will be analyzed throughout the test. Peak oxygen uptake (VO_2peak_) will be defined as the highest value reached during the test.

### Study intervention

After baseline evaluations, all patients will be instructed to present to the laboratory, three times per week for 2 weeks, for completion of the intervention stage of the study protocol, as follows:

- Day 1: Placement of the continuous glucose monitoring system. Patients should arrive at 8:00 a.m. for device placement. Patients using beta blockers will be advised to discontinue their medication 24 hours before the next study visit.
- Day 2: Acute inspiratory muscle exercise protocol (2 % or 60 % of PImax). Patients should arrive at 10:00 a.m. and will be instructed to rest in the supine position for 10 min for collection of baseline glucose, continuous blood pressure, and heart rate measurements. Participants will then start controlled ventilation for 10 min. A 40-minute interval will be allowed to elapse before the start of acute inspiratory muscle loading. Pursuant to random group allocation, participants will receive loading to 2 % of PImax (low resistance, placebo load) for 10 min or to 60 % of PImax (high resistance, experimental load) until task failure. At the end of the exercise protocol, a 10-min recovery record will be obtained. Glucose levels will also be measured by capillary sample before and after the protocol reported above. Arterial oxygen saturation (SpO_2_) mean arterial pressure, end-tidal carbon dioxide, calf blood flow, and calf vascular conductance will be measured during all protocols.
- Day 3: Continuous glucose monitoring system removal. Patients will be instructed to return to the laboratory at noon for removal of the device.

One week following these interventions, all subjects will return to the study facility to repeat these procedures, but using another randomized load for inspiratory muscle exercises.

The experimental sessions will take place at the Hospital de Clínicas de Porto Alegre Exercise Pathophysiology Research Laboratory. All experiments will be performed in a temperature-controlled room and all subjects will be instructed to refrain from consuming caffeinated beverages and alcohol for at least 12 hours and from exercise for at least 24 hours prior to the evaluation. Continuous assessment of blood-glucose levels and blood pressure will be performed before, during, and after all protocols. Patients will be asked to follow their habitual diet and keep a detailed food intake record across the 3 days of continuous glucose monitoring system use.

### Glucose variability evaluation

Subjects will be admitted to the laboratory at 8:00 a.m., 24 hours before the acute inspiratory muscle exercise session, for placement of the continuous glucose monitoring system sensor (Enlite Glucose Sensor, Medtronic MiniMed Inc., Northridge, CA, USA). Glucose data are obtained every 5 min, providing a total of 288 readings per day.

Individuals will be instructed to perform at least four capillary blood-glucose measurements per day, before each meal, for calibration. The monitoring will be performed by the patients themselves, with a digital glucometer (Accu-Check Performa, Roche Diagnostics, Mannheim, Germany). Along with the glucometer, diaries will be delivered to patients, in which they should record the glucose values at each measurement and also details of all meals ingested during the period of continuous glucose monitoring.

Glucose variability will be evaluated using conventional and non-conventional analyses. Conventional analysis will be conducted using the statistical properties of the glycemic series classically used [16, 39, 40] and non-conventional analysis of glucose variability will be conducted by application of two methods to the glucose series, based on spectral analysis and symbolic dynamics. Spectral analysis is a linear method that allows quantification of the oscillatory components from a time series by autoregressive models, and is widely applied to heart rate and arterial pressure series [41]. Symbolic dynamics relies on the calculation of Shannon entropy of the distribution of patterns lasting three measures and the classification of frequent deterministic patterns lasting three measures, and distributes deterministic patterns of the group into four variations [42].

### Cardiovascular autonomic control evaluation

Cardiovascular autonomic control will be measured continually before the protocol (during a 10 min rest), during the controlled ventilation plus acute inspiratory muscle exercise protocol (2 % or 60 % of PImax), and throughout the recovery period, using a noninvasive blood pressure device (NIBP100D system; Biopac, Santa Barbara, CA, USA) with a general purpose amplifier module (DA100; Biopac), according to methodological guidelines provided by Sherwood *et al.* [43]. This system acquires the blood pressure wave at a sampling frequency of 1000 Hz through a cuff placed on the middle phalanx of the third finger. Beat-to-beat time series of systolic blood pressure and pulse interval will be constructed from blood pressure recording data; their variances correspond to heart rate variability and blood pressure variability, respectively. In addition, spectral analysis, using an autoregressive model, will be applied to estimate the center frequency and power of each relevant oscillatory component [41]. The spectral bands for human beings are defined as: very low frequency, 0.0 to 0.04 Hz; low frequency, 0.04 to 0.15 Hz; and high frequency, 0.15 to 0.40 Hz, as described elsewhere [41].

### Controlled ventilation

Individuals will be placed in the supine position for measurement of respiratory rate, heart rate, SpO_2_ by pulse oximetry, and mean arterial pressure, which will be measured using an automated sphygmomanometer (Dinamap 1846 SX/P, Critikon, Tampa, FL, USA) once per minute during the test. The glucose and blood pressure, calf blood flow (which will be measured by venous occlusion plethysmography (Hokanson TL-400, Bellevue, WA, USA)) will be recorded continuously.

Inspiratory volume will be assessed using a pneumotachograph (Pneumotach3700 series, Hans Rudolph, Kansas City, MO, USA). The end-tidal carbon dioxide concentration will be measured by oxycapnography (Takaoka Oxicap, São Paulo, Brazil) and maintained at eupneic levels during both protocols via addition of CO_2_ to the inspiratory circuit. Participants will be instructed to maintain a breathing frequency of 15 breaths per min and a duty cycle (*T*_I_/*T*_Tot_) of 0.7 by listening to an audio signal with distinct inspiratory and expiratory tones, and visual feedback will be displayed.

### Acute inspiratory muscle exercise

This protocol was developed by Dempsey *et al.* [44] to reduce diaphragm blood flow and cause fatigue. During the protocol, individuals will breathe continuously through a two-way valve (Hans Rudolph, 2600 series, Shawnee, KS, USA) with low resistance (2 % of PImax) connected to an inspiratory resistance obtained by a threshold inspiratory muscle trainer (DHDInspiratory Muscle Trainer, Chicago, IL) or, for higher inspiratory pressures (60 % of PImax), to a POWERbreathe® inspiratory muscle trainer (Southam, UK). Inspiratory pressure will be continuously recorded and displayed on a computer screen shared through a television, providing visual feedback; the 10-point Borg scale [45] will be used to access inspiratory effort at task failure. Inability to maintain breathing will be defined as a reduction of PImax to less than 80 % of the prescribed value during three consecutive breaths [38]. For the placebo experiments conducted at 2 % of PImax, measurements will cease at 10 min. The sequence of experiments, induction of the inspiratory muscle metaboreflex (60 % of PImax), or allocation to the placebo experiment (2 % of PImax), will be randomized, and experiments will be conducted 1 week apart.

In both protocols, baseline data will be collected during 10 min of spontaneous breathing. After this period, a controlled ventilation protocol will be initiated, and individuals instructed to maintain a breathing frequency of 15 breaths per min and a duty cycle (*T*_I_/*T*_Tot_) of 0.7 by listening to an audio signal with distinct inspiratory and expiratory tones for 10 min. Calf blood flow will be measured by venous occlusion plethysmography (Hokanson, TL-400, Bellevue, WA, USA) every 10 s in the non-dominant lower limb. Calf vascular conductance will be calculated as calf blood flow divided by mean arterial pressure [46]. Blood pressure will be monitored once a minute using an automated monitor (Dinamap, USA), and heart rate will be monitored continuously throughout the protocol.

### Ethics and data protection issues

Participation will be voluntary, and all ethical principles of confidentiality and data protection will be followed. All subjects will have to sign a standardized informed consent form prior to participating in the study. All procedures will be explained to participants, and information about the aim, design, potential risks and benefits, and all relevant details of the study will be provided in the informed consent form. The data obtained by the study will be available to the participant and to any other authorized person, and may be used anonymously for academic scientific purposes. The study protocol was approved by the Institutional Review Board of Hospital de Clínicas de Porto Alegre, Brazil (number 14–0194) and registered at ClinicalTrials.gov (NCT02292810).

### Safety assessment

Data on all adverse events will be collected and included in medical reports, which will be forwarded to the health authorities. To ensure patient safety, if any adverse events occur at any time during the study, participants will be monitored for up to 4 weeks after study exit.

### Statistical analysis

Data will be expressed as means and standard deviations or medians and interquartile ranges for nonparametric variables. Two-way analysis of variance (ANOVA) for repeated measures will be used to evaluate averages before and after acute inspiratory muscle exercise, comparing the placebo (2 % of PImax) and intervention (60 % of PImax) loads. Pearson correlation coefficients will be used to evaluate the association between components of cardiovascular autonomic control and glucose levels. For nonparametric variables, Spearman correlation will be used. The statistical power will be 80 % and differences will be considered significant for *P* < 0.05. All analyses will be performed using SPSS Statistics for Windows, version 20.0 (IBM Corp., Armonk, NY, USA).

## Discussion

Exercise programs have traditionally emphasized aerobic exercise, because of its well-known cardiovascular [47], metabolic [48], and autonomic benefits in type 2 diabetes [24, 49]. Resistance training also has favorable effects on muscle strength, abdominal fat, insulin sensitivity [22], and blood pressure [21]. Furthermore, combined exercise is also beneficial for metabolic parameters [19, 20] and glucose variability in subjects with type 2 diabetes [18]. However, as not all patients tolerate conventional exercise and some find it difficult to adhere to fitness programs, alternatives must be developed to enable these patients to obtain the benefits of physical exercise. Inspiratory muscle training is a novel exercise modality that has demonstrated impressive beneficial effects on inspiratory muscle strength and resistance [29, 50] and in reduction of glucose variability [30]. However, given the relatively small sample size of our preliminary study [30], we planned to examine this question in greater depth through a new study with a larger sample of patients with type 2 diabetes, seeking to confirm that a single bout of inspiratory muscle exercise may provide a benefit similar to that achieved with aerobic exercise on glucose levels and glucose variability. In brief, this study will be conducted to evaluate glucose levels, glucose variability, and cardiovascular autonomic responses at rest, during, and after a session of acute inspiratory muscle exercise performed under two resistance loads (low, 2 % of PImax; high, 60 % of PImax).

## Trial status

The trial is currently in the recruitment phase. Each patient is scheduled to attend nine hospital visits, and the total data collection time will be 10 months.

## Abbreviations

ANOVA: analysis of variance
CONSORT: Consolidated Standards of Reporting Trials
HbA1c: glycated hemoglobin
PImax: maximal inspiratory pressure
SpO_2_: oxygen saturation
VO_2_peak: peak oxygen uptake.
